# Role of a novel tetrodotoxin-resistant sodium channel in the nitrergic relaxation of rattlesnake corpus cavernosum

**DOI:** 10.1186/1471-2210-11-S1-O26

**Published:** 2011-08-01

**Authors:** Gilberto De Nucci

**Affiliations:** 1 Department of Pharmacology, Faculty of Medical Sciences, UNICAMP, Campinas, SP, Brazil

## Introduction

Coitus in snakes may last up to 28 hours; however, the mechanisms involved are unknown.

## Aim

To evaluate the relevance of the nitric oxide (NO)-cyclic guanosine monophosphate (cGMP)-phosphodiesterase type 5 (PDE5) system in snake corpus cavernosum reactivity.

## Methods

Hemipenes were removed from anesthetized South American rattlesnakes (*Crotalus durissus terrificus*) and studied by light and scanning electronic microscopy. Isolated *Crotalus* corpora cavernosa (CCC) were dissected from the non-spiny region of the hemipenises, and tissue reactivity was assessed in organ baths.

## Main outcome measures

Cumulative concentration-response curves were constructed for acetylcholine (ACh), sodium nitroprusside (SNP), 5-cyclopropyl-2-[1-(2-fluorobenzyl)-1H-pyrazolo[3,4-b]pyridine-3-yl]pyrimidin-4-ylamine (BAY 41-2272), and tadalafil in CCC precontracted with phenylephrine. Relaxation induced by electrical field stimulation (EFS) was also done in the absence and presence of Nw nitro-L-arginine methyl ester (L-NAME;100 µM), 1H-[1, 2, 4] oxadiazolo[4,3-a]quinoxalin-1-one (ODQ; 10 µM) and tetrodotoxin (TTX; 1 µM).

## Results

The hemipenes consisted of two functionally concentric corpora cavernosa, one of them containing radiating bundles of smooth muscle fibers (confirmed by a-actin immunostaining). Endothelial and neural nitric oxide synthases were present in the endothelium and neural structures, respectively; whereas soluble guanylate cyclase and PDE5 were expressed in trabecular smooth muscle. ACh and SNP relaxed isolated CCC, with the relaxations being markedly reduced by L-NAME and ODQ, respectively. BAY 41-2272 and tadalafil caused sustained relaxations with potency (pEC50) values of 5.84 +0.17 and 5.10 + 0.08 (N = 3–4), respectively. In precontracted CCC, EFS caused frequency-dependent relaxations that lasted three times longer than those in mammalian CC. Although these relaxations were almost abolished by either L-NAME or ODQ, they were unaffected by TTX. In contrast, EFS-induced relaxations in marmoset CC were abolished by TTX.

## Conclusions

Rattlesnake CC relaxation is mediated by the NO-cGMP-PDE5 pathway in a manner similar to mammals. The novel TTX-resistant Na channel identified here may be responsible for the slow response of smooth muscle following nerve stimulation and could explain the extraordinary duration of snake coitus.

